# An institution-wide faculty mentoring program at an academic health center with 6-year prospective outcome data

**DOI:** 10.1017/cts.2019.412

**Published:** 2019-10-07

**Authors:** Heather Bonilha, Madison Hyer, Edward Krug, Mary Mauldin, Barbara Edlund, Bonnie Martin-Harris, Perry Halushka, Jacqueline McGinty, Joann Sullivan, Kathleen Brady, Dayan Ranwala, Kathie Hermayer, Jillian Harvey, Rechelle Paranal, Joseph Gough, Gerard Silvestri, Marc Chimowitz

**Affiliations:** 1 Department of Health Science and Research, Medical University of South Carolina, Charleston, SC, USA; 2 Department of Otolaryngology – Head and Neck Surgery, Medical University of South Carolina, Charleston, SC, USA; 3 Department of Public Health Sciences, Medical University of South Carolina, Charleston, SC, USA; 4 Department of Regenerative Medicine and Cell Biology, Medical University of South Carolina, Charleston, SC, USA; 5 Department of Library Science and Informatics, Medical University of South Carolina, Charleston, SC, USA; 6 College of Nursing, Medical University of South Carolina, Charleston, SC, USA; 7 Department of Communication Sciences and Disorders, Northwestern University, Evanston, IL, USA; 8 Departments of Pharmacology and Medicine, Medical University of South Carolina, Charleston, SC, USA; 9 Department of Neuroscience, Medical University of South Carolina, Charleston, SC, USA; 10 Office of Research, University of Alaska, Anchorage, AK, USA (previously Department of Library Science and Informatics, Medical University of South Carolina, Charleston, SC, USA; 11 Department of Psychiatry and Behavioral Sciences, Medical University of South Carolina, Charleston, SC, USA; 12 Department of Medicine, Medical University of South Carolina, Charleston, SC, USA; 13 Department of Healthcare Leadership and Management, Medical University of South Carolina, Charleston, SC, USA; 14 Department of Neurology, Medical University of South Carolina, Charleston, SC, USA

**Keywords:** Faculty mentoring, career development, career satisfaction, faculty turnover

## Abstract

**Background:**

There is discontent and turnover among faculty at US academic health centers because of the challenges in balancing clinical, research, teaching, and work–life responsibilities in the current healthcare environment. One potential strategy to improve faculty satisfaction and limit turnover is through faculty mentoring programs.

**Methods:**

A Mentor Leadership Council was formed to design and implement an institution-wide faculty mentoring program across all colleges at an academic health center. The authors conducted an experimental study of the impact of the mentoring program using pre-intervention (2011) and 6-year (2017) post-intervention faculty surveys that measured the long-term effectiveness of the program.

**Results:**

The percent of faculty who responded to the surveys was 45.9% (656/1428) in 2011 and 40.2% (706/1756) in 2017. For faculty below the rank of full professor, percent of faculty with a mentor (45.3% vs. 67.1%, *P* < 0.001), familiarity with promotion criteria (81.7% vs. 90.0%, *P* = 0.001), and satisfaction with department’s support of career (75.6% vs. 84.7%, *P* = 0.002) improved. The percent of full professors serving as mentors also increased from 50.3% in 2011 to 68.0% in 2017 (*P* = 0.002). However, the percent of non-retiring faculty considering leaving the institution over the next 2 years increased from 18.8% in 2011 to 24.3% in 2017 (*P* = 0.02).

**Conclusions:**

Implementation of an institution-wide faculty mentoring program significantly improved metrics of career development and faculty satisfaction but was not associated with a reduction in the percent of faculty considering leaving the institution. This suggests the need for additional efforts to identify and limit factors driving faculty turnover.

## Introduction

The current healthcare and research funding environments present many challenges to faculty at academic health centers [1]. Clinicians are under considerable pressure to increase clinical activities to maintain revenues, and researchers are seeking grant funding in an extremely competitive funding environment. Meanwhile, administrative and regulatory demands continue to grow. These circumstances have made it increasingly difficult for faculty to find time to teach, maintain scholarly productivity, and balance work and family life [2–5].

These challenging times have led to widespread discontent among faculty nationwide, and there are concerning trends in the high rates of faculty burnout and turnover at academic health centers [2,3,6–10]. In a recent survey by the Association of American Medical Colleges (AAMC) of 35 medical schools in the USA, almost a third of non-retiring faculty members were planning to leave or were considering leaving their medical schools in the next 2 years [10]. Compounding the loss of existing faculty is the fact that the pipeline of new clinical and translational researchers is diminishing [11,12].

One potential strategy to improve faculty satisfaction and lower the number of faculty leaving academic health centers is to improve faculty development and mentoring [13]. Studies focused on subgroups of faculty (e.g., minority faculty, female faculty, newly hired faculty) have shown that mentoring positively impacts the following outcomes for mentees: publications, grants and promotion [14–17], career satisfaction [15], feeling valued and supported by the institution [15,18], professional networking [19], and self-efficacy related to attaining career goals [20,21]. However, there are no studies with prospective outcome data on the effectiveness of an institution-wide mentoring program at an academic health center. This paper describes the development of such a program to improve mentoring at a stand-alone medical university by setting institutional philosophy and by providing the infrastructure to support mentoring across campus. The outcome of the program was evaluated using a prospective, pre- and post-assessment study design.

## Materials and Methods

### Medical University of South Carolina (MUSC) Campus-Wide Mentoring Program

In 2010, meetings between the leadership of MUSC’s South Carolina Clinical and Translational Research Institute (SCTR), the Provost, and the Deans of all six colleges, led to the decision to develop a University Mentor Leadership Council. This council was formed under the umbrella of SCTR, which is funded by an NIH Clinical and Translational Science Award. The council was charged with planning, developing, and coordinating mentoring programs across all colleges at MUSC, a stand-alone academic health center. As part of those activities, the council worked with leadership in all colleges (e.g., Associate Deans of Faculty Development) to strengthen mentoring activities throughout the institution. The goals of the council were to increase the number of junior faculty being mentored, enhance the quality of mentoring, and increase faculty satisfaction with career development.

The council was composed of senior mentors from all six MUSC colleges (Medicine, Nursing, Dentistry, Health Professions, Pharmacy, and Graduate Studies) and three mentees, two of whom were graduates of the SCTR KL2 scholars program. The initial activity of the council in 2010 was to develop a framework for departmental mentoring plans that were approved by the Provost and Deans of all the colleges for implementation in every department on campus. This document called Best Practices and Recommendations for Departmental Mentoring and Career Development Plans for Faculty was to be used by department chairs and assigned mentor champions (senior faculty who were responsible for leading the mentoring program in each department) as a blueprint to develop a new department mentoring program or enhance an existing program.

This document includes sections on: individual development plans; mentoring agreements; promotion and tenure; documentation of career development; resources for faculty development; hiring of new faculty and initial mentoring; developing, training, and rewarding mentors within the department; metrics of successful mentoring; and role of chairs, department promotion committees, and Deans. The document, which was updated once in 2016, is provided in the online Appendix.

All faculty at the level of associate professor or lower rank were included in this mentoring initiative regardless of focus (clinical, research, or education focus or combinations thereof) or tenure track (both tenure-track and nontenure track faculty were included). Details on how mentors were assigned are provided in the document in the online Appendix. In brief, junior and mid-level faculty who did not already have a mentor met with their department chairs or department mentor champions to identify potential mentors. The mentors were typically senior faculty in the same department but occasionally were in another department depending on the mentee’s needs. The junior and mid-level faculty members then contacted the senior faculty to request them to become their mentors. This was usually finalized in a subsequent meeting between the junior and senior faculty members.

Once the mentoring plans were implemented in each department, it was left to the leadership of each college to monitor the progress of faculty mentoring. In the College of Medicine, the largest college on campus, this was accomplished by convening quarterly group meetings of the mentor champions in each department to discuss challenging issues and their solutions. These meetings are overseen by the Associate Deans of Faculty Development in the college.

**Fig. 1.**
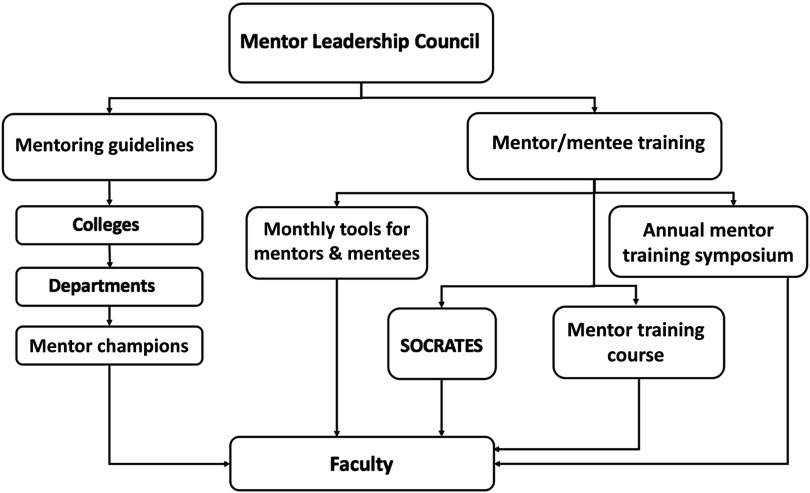
Conceptual model of the mentoring program showing key elements.

After developing the Best Practices document, the council initiated other mentoring activities that are available to all faculty. These include: (1) a monthly Tools for Mentors and Mentees series, which consists of a presentation by a faculty member with expertise in a mentoring topic (e.g., balancing work and life, developing a teaching portfolio, preparing grant budgets) (attendance, which is monitored, is 20–50 faculty depending on the topic), (2) an annual SCTR Mentorship Training Symposium that focuses on a particular theme (e.g., mentoring in team science, promotion, communication skills, negotiation skills) (attendance 80–120 faculty), (3) the Society for Research and Translational Early Scientists that meets twice per month to provide faculty an opportunity to present their research to senior faculty and peers (attendance 15–30 faculty), and (4) a Mentor Training Course offered twice per year to faculty who wish to improve their mentoring skills (attendance limited to 15 faculty per course). Modeled after the University of Wisconsin’s mentor training program for clinical and translational researchers [22], this four-session course focuses on six essential mentoring competencies: aligning expectations, maintaining effective communication, promoting professional development, fostering independence, addressing equity and inclusion, and assessing understanding. Refer to Fig. 1 for a conceptual model showing the key elements of the mentoring program.

### Career Development and Faculty Mentoring Surveys

All MUSC faculty were sent the career development and mentoring survey in 2011, prior to the implementation of the mentoring program in each department, and in 2017, after the mentoring program had been fully functioning for several years. Both surveys were identical and conducted online (Survey monkey in 2011 and Redcap in 2017) and were anonymous. Faculty were reminded twice, via email, about the survey after the initial link was provided in both 2011 and 2017. The survey was designed to assess the overall impact of the mentoring program at an institutional level and was not intended to evaluate the outcomes from the one-on-one mentoring relationships that developed as a result of the program (e.g., research productivity of mentees, promotion of mentees, etc.). Survey questions were developed through an iterative process by the Mentor Leadership Council, which includes a multidisciplinary team with expertise in mentorship, career development, and academic leadership. Questions were assessed and piloted by the council for content, clarity, and comprehensiveness related to the program objectives [23]. The survey consisted of questions related to: college and department affiliations; career development and satisfaction; mentoring activities (e.g., participation in the program, frequency and duration of mentoring meetings, satisfaction across multiple mentoring domains); barriers for the mentee and mentor; interest in mentor training opportunities; demographic questions (optional if faculty had concerns about anonymity); and considering leaving MUSC in the next 2 years. A copy of the 2017 survey is available in the online Appendix.

### Data Analysis

All demographic variables and survey responses were collected as categorical variables and are presented as frequency (%) and with 95% confidence intervals (CIs), where appropriate. Comparisons of survey results between 2011 and 2017 were performed using chi-square tests or, where appropriate, Mantel–Haenszel chi-square or Fisher’s exact tests to test the overarching hypothesis that the amount of faculty mentoring and metrics related to career development and satisfaction would improve as a result of the mentoring program. We considered a relative increase of at least 10% in these metrics from 2011 to 2017 to be a meaningful difference. Using the 2017 data, we assessed the predictive value of demographic and mentoring variables with satisfaction with department support and considering leaving the institution in the next 2 years for faculty below the rank of full professor. For each of these two outcomes, potential predictors were assessed for bivariate associations and those that were found statistically significant (*P* < 0.05) were entered into a multivariable logistic regression model. To ensure adequate model fit, the Hosmer–Lemeshow goodness-of-fit tests were performed for each of the models. Results of the logistic regression are presented as odds ratios (ORs) and 95% CIs. Statistical significance was assessed at *P* = 0.05. All analyses were performed using SAS v9.4 ©.

## Results

### Respondent Demographics

The percent of faculty who responded to the surveys was 45.9% (656/1428) in 2011 and 40.2% (706/1756) in 2017. The majority of the respondents were from the College of Medicine in both 2011 (496/656; 75.6%) and in 2017 (508/706; 71.9%). These numbers closely approximate the percentage of faculty at MUSC who are in the College of Medicine (≈75%). Table 1 shows the demographics of the respondents in 2011 and 2017. The only demographics that were significantly different between the 2011 and 2017 respondents were: more new hires (at MUSC for <3 years) and fewer long-time faculty (at MUSC for more than 15 years) in 2017. Proportions between groups for other demographic variables did not differ significantly.

**Table 1.** Demographics of the faculty responding to the survey

	2011	2017	
Count (%)	*N* = 656	*N* = 706	*P*-value
Rank			0.08
Instructor/Other	33/523 (6.3%)	39/523 (7.5%)	
Assistant	224/523 (42.8%)	184/523 (35.2%)	
Associate	122/523 (23.3%)	144/523 (27.5%)	
Professor	144/523 (27.5%)	156/523 (29.8%)	
Tenure			0.82
Nontenure track	178/565 (31.5%)	176/532 (33.1%)	
Tenure track	234/565 (41.4%)	219/532 (41.2%)	
Tenured	153/565 (27.1%)	137/532 (25.8%)	
Age			0.82*
Younger than 30	9/543 (1.7%)	5/491 (1%)	
30–39	151/543 (27.8%)	145/491 (29.5%)	
40–49	141/543 (26%)	136/491 (27.7%)	
50–59	145/543 (26.7%)	109/491 (22.2%)	
60 or older	97/543 (17.9%)	96/491 (19.6%)	
Gender			0.12
Males	320/555 (57.7%)	273/516 (52.9%)	
Females	235/555 (42.3%)	243/516 (47.1%)	
Race			0.09
White	466/528 (88.3%)	420/459 (91.5%)	
Non-white	62/528 (11.7%)	39/459 (8.5%)	
Years at MUSC			0.04*
3 years or less	127/570 (22.3%)	152/535 (28.4%)	
4–7 years	142/570 (24.9%)	115/535 (21.5%)	
8–15 years	127/570 (22.3%)	133/535 (24.9%)	
More than 15 years	174/570 (30.5%)	135/535 (25.2%)	

Comparisons of 2011 and 2017 data were performed using chi-square tests except for those variables indicated by a * for which a Mantel–Haenszel chi-square test was used. Abbreviations: MUSC, Medical University of South Carolina.

### Familiarity of Faculty with Department Mentoring Plans

The percent of faculty who were familiar with their departmental mentoring plans almost doubled from 2011 (36.7%) to 2017 (69.7%) (*P* < 0.001). There was some variability in the response to this question in different colleges and departments. In the College of Medicine, the 2017 survey showed that at least 75% of faculty in 16 of 23 (69.5%) departments were familiar with their department plan (six departments had 100% familiarity). In the other seven departments, fewer than 75% of the faculty were familiar with the department plan. In the Colleges of Health Professions and Nursing, 76.0%–86.3% of their faculty were familiar with their department plan.

### Number of Mentors and Mentees

Comparisons of the faculty responses to questions related to number of mentors and mentees between 2011 and 2017 are shown in Table 2. There were significantly more instructors and assistant professors with a mentor in 2017 (67.9%) than in 2011 (51.4%) (*P* < 0.001), but a higher percentage of these faculty had more than one mentor in 2011 than in 2017 (36.3% vs. 24.7%, *P* = 0.04). The percent of associate professors with mentors was also significantly higher, doubling in 2017 (66.0%), compared with (32.2%) in 2011 (*P* < 0.001). The percent of full professors with a mentoring role was higher in 2017 (67.9%) than in 2011 (50.3%) (*P* = 0.002) but their number of mentees did not increase (*P* = 0.80) (see Table 2).

**Table 2.** Number of mentees and mentors

	2011	2017	*P*-value
Instructors and Assistant Professors	*N* = 257 (frequency & 95% CI)	*N* = 216 (frequency & 95% CI)	
Do you have a mentor? Yes	129/251 (51.4%)	146/215 (67.9%)	<0.001
	(45.2%-57.5%)	(61.3%-73.7%)	
How many mentors?			0.04
1	79/124 (63.7%)	110/146 (75.3%)	
2 or more	45/124 (36.3.0%)	36/146 (24.7%)	

Associate Professors	*N* = 122	*N* = 124	
Do you have a mentor? Yes	38/118 (32.2%)	95/144 (66.0%)	<0.001
	(24.4%-41.1%)	(57.9%-73.2%)	
How many mentors?			0.69
1	26/37 (70.3%)	70/95 (73.7%)	
2 or more	11/37 (29.7%)	25/95 (26.3%)	
Do you serve as a mentor? Yes	20/121 (16.5%)	31/143 (21.7%)	0.29
	(10.86%-24.3%)	(15.6%-29.1%)	
How many mentees?			0.07*
1	13/19 (68.4)	12/31 (38.7%)	
2	5/19 (26.3%)	16/31 (51.6%)	
3	1/19 (5.3%)	2/31 (6.5%)	
4	0/19 (0.0%)	0/31 (0.0%)	
5	0/19 (0.0%)	1/31 (3.2%)	

Full Professors	*N* = 144	*N* = 156	
Do you aerve as a mentor? Yes	72/143 (50.3%)	106/156 (67.9%)	0.002
	(42.2%-58.4%)	(60.2%-74.7%)	
How many mentees?			0.80*
1	22/69 (31.9%)	27/106 (25.5%)	
2	17/69 (24.6%)	28/106 (26.4%)	
3	5/69 (7.3%)	18/106 (17.0%)	
4	10/69 (14.5%)	10/106 (9.4%)	
5	15/69 (21.8%)	23/106 (21.7%)	

Comparisons of 2011 and 2017 data were performed using chi-square tests except for those variables indicated by a * for which a Mantel–Haenszel chi-square test was used.

### Mentoring Activities

The frequency and length of mentoring meetings as reported by the mentees significantly changed in 2017 compared with 2011 (Table 3): in 2011, 57.1% of mentees met with their mentors at least monthly compared with 45.5% in 2017; however, the most common meeting lengths were 30 minutes (53.1%) in 2011 compared with an hour (53.2%) in 2017.

**Table 3.** Mentoring activities & satisfaction

	2011	2017	
Instructor, Assistant and Associate Professors	*N* = 379 (frequency & 95% CI)	*N* = 360 (frequency & 95% CI)	p-Value
Familiar with department mentoring plan	139/379 (36.7%)	251/360 (69.7%)	<0.001
	(31.9%-41.6%)	(64.7%-74.2%)	
Familiar with promotion criteria	308/377 (81.7%)	324/360 (90.0%)	0.001
	(77.4%-85.2%)	(86.4%-92.7%)	
Satisfied with departmental support	275/364 (75.6%)	304/359 (84.7%)	0.002
	(70.8%-79.6%)	(80.5%-88.0%)	
Satisfied with career progress	316/376 (84.0%)	315/359 (87.7%)	0.15
	(79.9%-87.4%)	(83.9%-90.7%)	
Do you have a mentor? Yes	167/369 (45.3%)	241/359 (67.1%)	<0.001
	(40.2%-50.3%)	(62.1%-71.7%)	
How frequently do you meet?			0.015
Weekly	42/163 (25.8%)	39/235 (16.6%)	
Monthly	51/163 (31.3%)	68/235 (28.9%)	
Quarterly	27/163 (16.6%)	54/235 (23.0%)	
Every 6 months	19/163 (11.7%)	35/235 (14.9%)	
Annually	24/163 (14.7%)	39/235 (16.6%)	
What is the length of your meeting?			0.09
30 minutes	85/160 (53.1%)	97/233 (41.6%)	
60 minutes	68/160 (42.5%)	124/233 (53.2%)	
90 minutes	4/160 (2.5%)	11/233 (4.7%)	
120 minutes	3/160 (1.9%)	1/233 (0.4%)	
Satisfaction with research mentoring^*^	128/158 (81.0%)	182/218 (83.5%)	0.54
	(74.1%-86.4%)	(77.9%-87.8%)	
Satisfaction with teaching/education mentoring^*^	99/122 (81.2%)	165/190 (86.8%)	0.17
	(73.2%-87.1%)	(81.2%-90.9%)	
Satisfaction with clinical mentoring^*^	68/83 (81.9%)	136/151 (90.1%)	0.07
	(72.1%-88.8%)	(84.1%-93.9%)	
Satisfaction with administration mentoring^*^	90/113 (79.7%)	145/176 (82.4%)	0.56
	(71.2%-86.1%)	(76.0%-87.3%)	
Satisfaction with career development mentoring^*^	122/157 (77.7%)	197/233 (84.6%)	0.09
	(70.5%-83.5%)	(79.3%-88.6%)	
Satisfaction with balancing work and personal life mentoring^*^	88/128 (68.8%)	148/193 (76.7%)	0.11
	(60.2%-76.1%)	(70.2%-82.1%)	

Full Professors	*N* = 144	*N* = 156	
Mentoring is a very satisfying part of my job	70/71 (98.6%)	101/105 (96.2%)	0.65
	(91.7%–>99.9%)	(90.3%-98.8%)	
My department values my roles as a mentor	62/71 (87.3%)	87/103 (84.5%)	0.67
	(77.4%-93.4%)	(76.1%-90.3%)	
Mentoring has made my mentee more productive	68/70 (97.1%)	99/104 (95.2%)	0.70
	(89.5%-99.8%)	(88.9%-98.2%)	

* Faculty were considered satisfied if they indicated “satisfied” or “very satisfied” on the survey.

### Satisfaction with Mentoring

Satisfaction of instructors, assistant professors, and associate professors with mentoring across multiple domains (research, clinical, teaching, administration, career development, work–life balance) remained stable for all domains from 2011 to 2017 (Table 3) with no statistically significant increases or decreases. Almost all full professors serving as mentors reported that mentoring was a very satisfying part of their jobs (98.6% in 2011 and 96.2% in 2017) and contributed to the productivity of the mentees (97.1% in 2011 and 95.2% in 2017), and most felt valued as mentors by their departments (87.3% in 2011 and 84.5% in 2017).

### Barriers to Mentoring

Mentee barriers that were similar in 2011 versus 2017 were insufficient time to be a mentee (37.7% vs. 41.6%, *P* = 0.44) and insufficient pool of mentors (60.1% vs. 52.3%, *P* = 0.12). However, significantly fewer mentees considered mentoring resources to be insufficient in 2017 (38.1%) versus 2011(56.5%) (*P* < 0.001). Mentor barriers in 2011 and 2017 were insufficient time to be a mentor (52.8% vs. 47.6%, *P* = 0.50), insufficient mentoring resources (55.7% vs. 54.4%, *P* = 0.86), insufficient salary support for mentoring (71.6% vs. 64.0%, *P* = 0.38), and insufficient mentor training (44.3% vs. 48.0%, *P* = 0.69).

### Career Development and Career Satisfaction

For faculty below the rank of full professor, there was a significant increase in the following metrics from 2011 to 2017: familiarity with their college’s promotion criteria (81.7% vs. 90.0%, *P* = 0.001) and satisfaction with departmental support of their careers (75.6% vs. 84.7%, *P* = 0.002) both meaningfully increased (Table 3).

Variables associated with satisfaction with departmental support by faculty below the rank of full professor in 2017 are shown in Table 4. Having a mentor, female gender, and familiarity with promotion criteria were significantly associated with satisfaction with departmental support in univariate analyses. In multivariate analysis, having a mentor and female gender remained significant (Table 4).

**Table 4.** Variables associated with faculty^*^ satisfaction with their department’s support and considering leaving the institution in the next 2 years

Variable	Comparison	*N* (%)	Bivariate p-Value	Multivariate p-Value	Odds ratio	CI
Variables associated with faculty satisfaction with their department’s support						
Have a mentor?	No	92/118 (78.0%)	0.014	0.006	Ref	Ref
	Yes	211/240 (87.9%)			2.23	1.17–4.26
Gender	Male	126/159 (79.3%)	0.001	0.003	Ref	Ref
	Female	167/182 (91.8%)			2.68	1.38–5.21
Familiar with promotion criteria?	No	26/36 (72.2%)	0.029	0.17	Ref	Ref
	Yes	278/323 (86.1%)			1.86	0.77–4.47
Variables associated with faculty considering leaving the institution in the next 2 years						
College	Other	10/86 (11.6%)	0.020	0.019	Ref	Ref
	Medicine	63/271 (23.3%)			2.73	1.18–6.33
Gender	Female	25/183 (13.7%)	0.003	0.017	Ref	Ref
	Male	42/159 (26.4%)			1.98	1.13–3.48

* Faculty included were instructors, assistant professors, and associate professors.

### Considering Leaving the Institution

Excluding faculty considering retiring, the percent of faculty considering leaving in the next 2 years was significantly higher in 2017 (136 of 560 (24.3%)) than in 2011 (120 of 637 (18.8%)) (*P* = 0.02). For the College of Medicine, by far the largest college on campus, the percent of faculty considering leaving in the next 2 years was 27.0% (113 of 419) in 2017 compared with 21.9% (106 of 484) in 2011 (*P* = 0.08). These faculty were considering positions at another academic health center (75.7%), private practice (19.1%), industry (12.5%), or a government agency (12.5%) (total > 100% because some faculty were considering multiple options).

Variables associated with faculty below the rank of full professor in 2017 who were considering leaving in the next 2 years are shown in Table 4. College of Medicine and male gender were the variables significantly associated with considering leaving in both univariate and multivariate analyses.

## Discussion

This mentorship program, which to our knowledge is the first institutional-wide mentoring program at an academic medical center, led to a large increase in the number of faculty below the rank of full professor with mentors from 2011 to 2017. The percent in 2017 (67.1%) is substantially higher than the 44% of faculty within 5 years of a first appointment who have a mentor as reported by the AAMC following a survey of 35 academic medical centers in the USA between 2013 and 2016 [10]. In general, the higher level of mentorship in 2017 at MUSC was achieved by substantially increasing the number of mentors and not increasing the mentoring load (number of mentees per mentor) of the senior faculty. The increase in the number of mentees and mentors in 2017 is indicative of widespread belief in the importance of mentoring following the initiation of our institution-wide mentoring program.

The substantial increase in the percentages of junior faculty being mentored and senior faculty serving as mentors did not result in lowering the quality of mentoring. In fact, satisfaction with mentoring across all domains (research, clinical, teaching, administration, career development, work–life balance) remained stable from 2011 to 2017 (Table 3). This is encouraging as it indicates the ability to provide quality mentorship to a large number of faculty. Measures of career development and satisfaction that increased significantly from 2011 to 2017 were familiarity with promotion criteria and satisfaction with departmental career support (Table 3). Multivariate analyses (Table 4) showed that increasing the number of faculty being mentored played an important role in faculty satisfaction with their department’s support.

It is disappointing that despite a substantial increase in mentoring activities and faculty satisfaction with their department’s support, a significantly higher percentage of faculty below the rank of full professor were considering leaving the institution in the next 2 years in 2017 (24.3%) compared with 2011 (18.8%). One of the factors that was significantly associated with faculty considering leaving in 2017 was an appointment in the College of Medicine (Table 4), which instituted a new faculty compensation plan in 2017. It is possible that this and other factors (e.g., new opportunities, family reasons, the challenge of work–life balance, physician burnout [6,7]) influenced some faculty to consider leaving [24,25]. These same factors may have influenced the 2017 survey results for questions related to barriers to mentoring, career development, and career satisfaction. Although the percent of non-retiring faculty considering leaving over the next 2 years increased since 2011, the percentages in 2017 (24.3% for all faculty; 27% for faculty in the College of Medicine) are still numerically lower than the 29% of non-retiring faculty who are considering leaving their medical schools over the next 2 years according to a recent AAMC survey [10].

Despite numerous improvements in mentoring metrics in 2017 compared with 2011, there are a number of areas in need of improvement: increasing the number of mentees that meet at least monthly with their mentors; improving the quality of mentoring; and decreasing barriers to being a mentee or mentor. While 45.5% of junior faculty met with their mentors at least monthly in 2017, 23% met quarterly, and 16.6% only met annually. Infrequent meetings do not reflect a strong mentoring relationship and the benefits of mentorship may not occur at this level of engagement. Mentee satisfaction was between 76.7% and 84.6% for the areas of mentoring related to research, administration, career development, and work–life balance in 2017 (Table 3), which provides areas on which to focus increased mentor training and resources. While barriers to mentorship tended to decrease from 2011 to 2017, many respondents still reported barriers such as lack of time, lack of available mentors, insufficient mentor salary support, and insufficient mentor training, indicating that more efforts in these areas can further increase the effectiveness of mentoring across campus. We have used this information to increase the visibility and advertisement of our mentoring resources and mentor training. We will also focus future mentoring-related activities on developing and distributing information to help mentors best use their time.

Study limitations: We present an institution-wide mentoring initiative with college- and department-led implementation of the best practice guide. This resulted in variations in the actual implementation and utilization of the best practices in different departments and colleges (see section “*Familiarity of Faculty with Department Mentoring Plans*” in Results). Nevertheless, in the absence of other institution-wide programs with similar goals, it is likely that the mentoring program played the primary role in improving metrics related to the amount of mentoring and the career development and satisfaction of our faculty as a whole. As in all survey studies, our data may reflect response bias. Faculty who respond to such surveys typically have a bimodal rationale for being interested, either they are highly positive of the survey topic or highly negative, which could have influenced the survey results. The cohort sampled in the 2011 survey was not identical to that sampled in 2017 because of changes in faculty over the 6-year study period, and this is reflected in the statistically significant differences in rank and time at MUSC of the respondents in 2017 compared with 2011 (Table 1). It is likely that some of the faculty did not receive the full value of the mentoring program, that is, have exposure to resources and training that other faculty who were at MUSC all 6 years received. This may indicate that our results undervalued the impact of the mentoring plan. Our assessment of mentoring and career development effectiveness was based on subjective assessments by the mentees and mentors rather than on more objective outcomes like publications, grants, and teaching assessments. This study evaluates a faculty mentoring program within a single stand-alone academic health center; therefore, results may not be generalizable to other academic health centers. However, the lessons learned from this program may be adapted by others who are developing mentorship programs at similar institutions.

## Conclusions

Implementation of an institution-wide faculty mentoring program at a stand-alone academic health center significantly and meaningfully increased the number of faculty with mentors and the number of full professors serving as mentors. The program also meaningfully improved a number of metrics related to career development (familiarity with promotion criteria and their departmental mentoring plan) and departmental support of their career. Nevertheless, the percent of faculty considering leaving the institution over the next 2 years increased (though remains below the national average). These findings suggest that while mentoring is important for faculty development and career satisfaction, it is insufficient for reducing potential faculty turnover. This implies the need for additional programs to identify and mitigate against factors contributing to high faculty turnover at academic health centers.
