# An institution-wide faculty mentoring program at an academic health center with 6-year prospective outcome data – ADDENDUM

**DOI:** 10.1017/cts.2020.4

**Published:** 2020-03-31

**Authors:** Heather Bonilha, Madison Hyer, Edward Krug, Mary Mauldin, Barbara Edlund, Bonnie Martin-Harris, Perry Halushka, Jacqueline McGinty, Joann Sullivan, Kathleen Brady, Dayan Ranwala, Kathie Hermayer, Jillian Harvey, Rechelle Paranal, Joseph Gough, Gerard Silvestri, Marc Chimowitz

In the above article by Bonilha et al. [1], online-only supplementary material was supposed to be included. The files have now been added online.

The publisher apologizes for this omission.
